# Supplementary material for: Annexin Induces Cellular Uptake of Extracellular Vesicles and Delays Disease in Escherichia coli O157:H7 Infection
Source: Microorganisms. 2021 May 26;9(6):1143. doi: 10.3390/microorganisms9061143 (PMC8228561; doi:10.3390/microorganisms9061143)
Supplement: Supplementary file 1 [file microorganisms-09-01143-s001.zip › microorganisms-1235640-supplementary.pdf]

Annexin induces cellular uptake of extracellular vesicles and delays disease in *Escherichia coli* O157:H7 infection

Ashmita Tontanahal, Ida Arvidsson, Diana Karpman

Supplementary methods

Detection of Shiga toxin 2 in mouse kidneys

Shiga toxin 2 (Stx2) detection in the kidneys of *Escherichia coli* O157:H7-infected mice sacrificed on day 5 was performed by immunofluorescence staining, Stx2-ELISA or mass spectrometry. Tissue was collected, fixed in 4% paraformaldehyde (Histolab Products, Askim, Sweden) and embedded in paraffin. Paraffin-embedded kidney sections were used for immunofluorescence staining, while proteins were extracted from paraffin-embedded kidney sections as previously described [1] and used for Stx2 ELISA and mass spectrometry.

Immunofluorescence

Paraffin embedded kidney sections were subjected to immunofluorescence staining as previously described [2]. with modifications to detect Stx2. Briefly, the sections were subjected to deparaffinization and a series of rehydration steps. They sections were then subjected to antigen retrieval and blocked with 5% BSA for 1 h at room temperature The sections were then incubated with camelid anti-Stx1 and Stx2 (1:100, List Biological Laboratories, Campbell, CA, USA) diluted in 1% BSA overnight at 4°C in a humid chamber, washed three times with PBS-Tween (Medicago, Uppsala, Sweden) and then incubated with goat anti-llama:FITC (1:2000, Invitrogen, Carlsbad, CA, USA) diluted in 1% BSA for 1 h at room temperature. The sections were washed again as above and mounted with ProLong Diamond Antifade Mountant with DAPI (Thermo Fischer Scientific, Rockford, IL, USA) and visualized using a Ti-E inverted fluorescence microscope equipped with a Nikon structured illumination microscopy module

(Nikon Instruments Inc., Tokyo, Japan) and imaged using NIS elements AR software v.5.11.01.

Stx2 ELISA

Camelid anti-Stx1 and Stx2 (0.5 mg/mL) diluted 1:6000 times in carbonate buffer (0.1 M, pH 9.6) was coated on a MaxiSorp nunc plate (Thermo Fischer Scientific) and incubated overnight at 4°C. The plate was washed three times with PBS-Tween and blocked with 1% BSA for 1 h at room temperature. The extracted proteins were loaded on to the plate and incubated overnight at room temperature. The plate was washed as above and incubated with mouse anti-Stx2 (1 μg/mL, clone 11E10, Santa Cruz Biotechnology, Dallas, TX, USA) diluted in 1% BSA for 1 h at room temperature. The plate was washed as above and incubated with goat anti-mouse IgG-HRP (1:1000, Dako, Glostrup, Denmark) in 1% BSA for 1 h at room temperature. The plate was washed and Super signal ELISA pico chemiluminescent substrate (Thermo Fischer Scientific) was added according to manufacturer's protocol. The chemiluminescent signal was detected at one-sec integration time in a Glomax Discover system (Promega, Madison, WI, USA). Stx2 (0.78-50 ng/mL, Phoenix Lab, Tufts Medical Centre, Boston, MA, USA) diluted in ammonium bicarbonate (because the protein extracts contained ammonium bicarbonate) was used as the standard.

Mass spectrometry

Tryptic digestion of Stx2

Pure Stx2 (4 μg) was subjected to tryptic digestion to determine its pattern of peptide peaks. Stx2 was reduced with 10 mM dithiothreitol for 30 min at 56°C followed by alkylation using 40 mM iodoacetamide for 30 min in the dark. The sample was digested with 0.1 μg trypsin (Sequencing Grade Modified Trypsin, Part No. V511A, Promega, Madison, WI, USA)

overnight at 37 °C. The digestion was stopped by addition of 5 μL 10% trifluoroacetic acid. The sample then underwent desalting using Ultra Microspin Silica C18 column (SUM SS18V, 3–30 μg capacity, The Nest Group Inc., Southborough, MA, USA). The sample was dried by centrifugal evaporator and resuspended with 40 μL of 0.1% formic acid/2% acetonitrile. Peptide concentration was measured at 215 nm using a DS-11 FX Spectrophotometer (DeNovix Inc, Wilmington, DE, USA). The digested Stx2 (0.6 μg), was injected to Liquid Chromatography with Tandem Mass Spectrometry (LC-MS/MS).

Sample preparation from paraffin-embedded kidney sections

Proteins extracted from sections where run on LC-MS/MS as previously described with modifications to detect Stx2 [1]. Protein extracted from an untreated control mouse was spiked with pure Stx2 (1, 10 and 100 ng) as a control. For detection of Stx2 in the samples, parallel reaction monitoring (PRM) assay was performed in order to target a specific protein in a complex sample. The raw acquired data was compared against sequences for Stx2A and Stx2B derived from *E. coli* O157:H7 (PATRIC 3.6.9, The PATRIC Bioinformation Resource Center).

Mass spectrometry data acquisition

The transition lists were created in Skyline v20.2 software (MacCoss Lab, Seattle, WA, USA). Primarily, high numbers of transitions, all possible y-ion series that match the criteria (from *m/z* > precursor 2 to last ion-1), were selected for each peptide at both 2+ and 3+ charge states.

The peptide analysis was performed on a Q Exactive HFX mass spectrometer (Thermo Fischer Scientific) connected to an Ultimate 3000 ultra-high-performance liquid chromatography system (Thermo Fischer Scientific). Peptides (1 μg) were loaded on a precolumn (Thermo Scientific, ID 75mm ´ 2 cm, column temperature 35°C) and then separated on an EASY-Spray

column (Thermo Fischer Scientific, ID 75mm ´ 25 cm, column temperature 45°C). A nonlinear gradient of buffer B (80% acetonitrile, 0.1% formic acid) in buffer A (aqueous 0.1% formic acid) was applied at a flow rate of 350 nL/min. For the gradient, the percentage of solvent B was increased from 10% to 30% in 75 min and then increased to 45 % in 15 min and then increased from 45% to 95% in 1 min and kept for 6 min. The solvent B was decreased to 3% in 1 min and kept for 9 min.

One full MS scan (resolution 120,000 at 200 *m/z*; mass range 350–1,400 *m/z*) was followed by inclusion-list driven MS/MS scans (resolution 30,000 at 200 *m/z*). The precursor ions were isolated with 1.2 *m/z* isolation width and fragmented using higher-energy collisional-induced dissociation at a normalized collision energy of 27. Charge state screening was enabled, and singly charged ions as well as precursors with a charge state above 6 were rejected. The dynamic exclusion window was set to 15 s. The automatic gain control was set to 3e6 for MS and 2e5 for MS/MS with ion accumulation times of 45 ms and 250 ms, respectively. The intensity threshold for precursor ion selection was set to 2e5. An inclusion list generated in Skyline was included in the PRM-method.

References

1. Hu, D.; Ansari, D.; Pawłowski, K.; Zhou, Q.; Sasor, A.; Welinder, C.; Kristl, T.; Bauden, M.; Rezeli, M.; Jiang, Y., et al. Proteomic analyses identify prognostic biomarkers for pancreatic ductal adenocarcinoma. *Oncotarget* **2018**, *9*, 9789-9807, doi:10.18632/oncotarget.23929.
2. Calderon Toledo, C.; Rogers, T.J.; Svensson, M.; Tati, R.; Fischer, H.; Svanborg, C.; Karpman, D. Shiga toxin-mediated disease in MyD88-deficient mice infected with *Escherichia coli* O157:H7. *Am. J. Pathol.* **2008**, *173*, 1428-1439, doi:10.2353/ajpath.2008.071218.

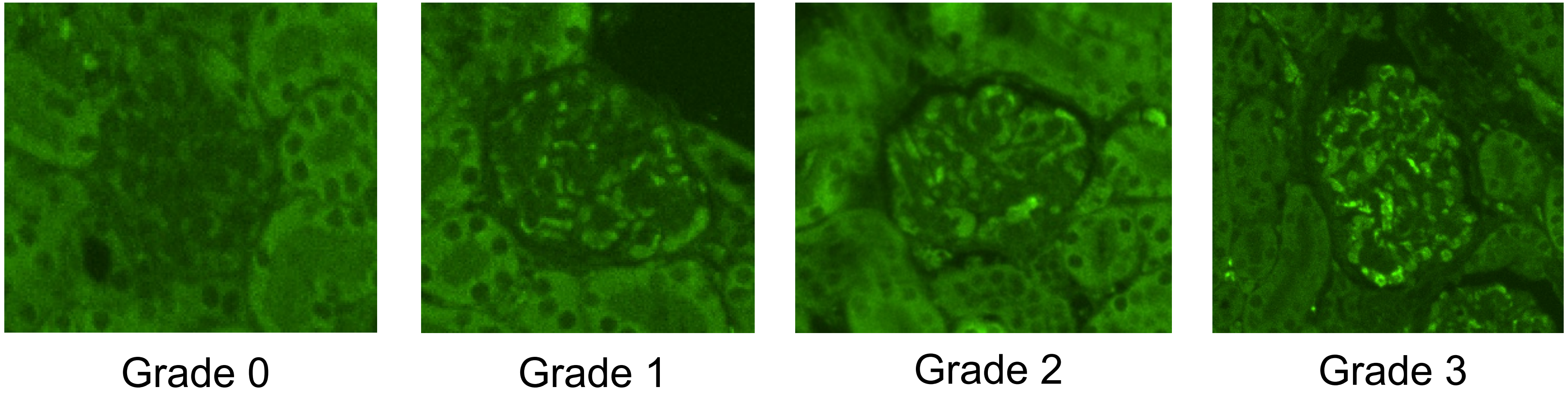

Figure S1: Grading template used to quantify glomerular fibrinogen deposition in murine kidney
Renal tissue from an *E. coli* O157:H7-infected mouse treated with PBS vehicle was analysed by immunofluorescence for fibrinogen deposition in glomeruli. The level of intensity was graded using a scoring system of no staining (0), low (1), medium (2), or high (3) intensity. This figure was used as the template for quantification shown in Figure S3.

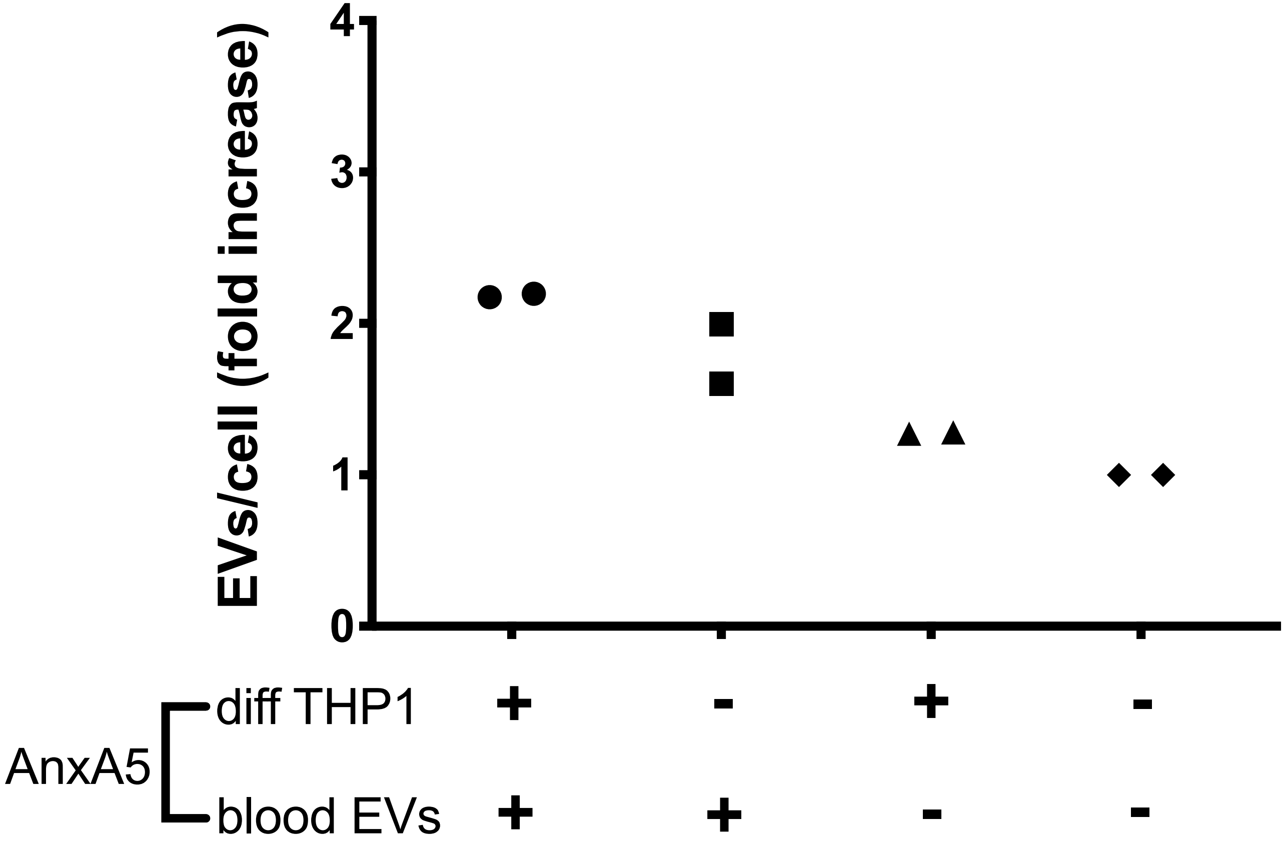

Figure S2: Uptake of Shiga toxin 2-positive extracellular vesicles by THP1 cells in the presence of annexin A5
Differentiated THP1 cells and blood cell-derived extracellular vesicles (EVs), shed after Shiga toxin 2 stimulation, were pre-incubated with or without annexin A5 (anxA5) and then co-incubated. The presence of anxA5, both on extracellular vesicles (EVs) and cells as well as only on EVs resulted in an increase in EV uptake by differentiated THP1 cells in comparison to the absence of anxA5 on both EVs and cells. EV uptake by cells is represented as relative to the sample in which neither cells nor EVs were pre-incubated with anxA5 (defined as 1) in order to account for experimental variability. Two experiments are shown.

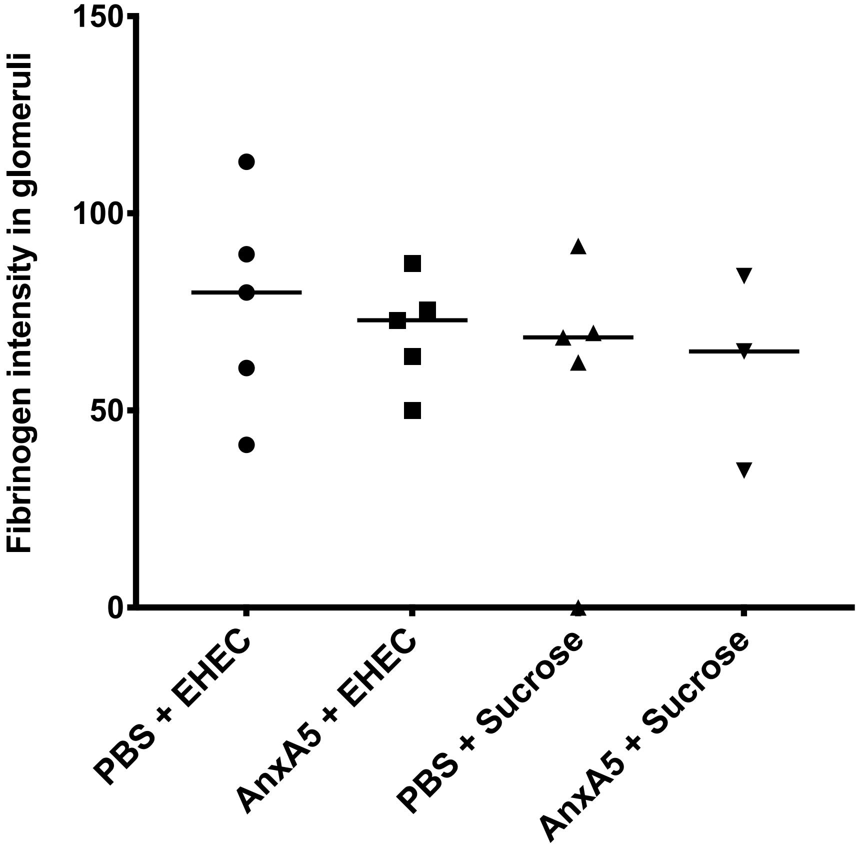

Figure S3: Fibrinogen deposition in murine kidneys
Kidney sections from *E. coli* O157:H7 infected mice treated with annexin A5 (anxA5) 500 µg/kg (n=5) or PBS vehicle (n=5) and uninfected mice treated with anxA5 500 µg/kg (n=3) or PBS vehicle (n=5) sacrificed on day 5, exhibited fibrinogen deposition in the glomeruli. The degree of fibrinogen staining intensity as per Figure S1 was multiplied by the number of glomeruli and the total level of intensity in each kidney section was calculated. No statistical significance was found. The bar represents the median.
